# Comment on ‘Muscle‐Specific Strength Better Predicts Physical Performance Decline Than Conventional Metrics: The I‐Lan Longitudinal Aging Study’ by Chien et al.

**DOI:** 10.1002/jcsm.70194

**Published:** 2026-01-25

**Authors:** Ricardo M. Lima, Anthony J. Blazevich

**Affiliations:** ^1^ Department of Physical Education University of Brasília Brasília Brazil; ^2^ Centre for Human Performance, School of Medical and Health Sciences Edith Cowan University Joondalup Western Australia Australia

We read with great interest the article by Chien et al. [1]. The authors are to be congratulated for elegantly addressing the association between muscle‐specific strength (MSS) and physical performance decline in community‐dwelling older adults. Their salient finding—that low MSS, assessed by handgrip strength (HGS) normalized to the lean mass of the dominant hand, independently predicts deterioration of physical performance and associates with adverse metabolic and inflammatory biomarkers—adds important evidence to the field and suggests that MSS may provide a more meaningful indicator of muscle health than conventional measures, including absolute HGS. The relevance of exploring MSS is underscored by the fact that the European Working Group on Sarcopenia in Older People (EWGSOP) [2] has incorporated muscle quality as a diagnostic component, often operationalized as the ratio of strength to muscle volume, and that this concept has been recently endorsed by the Global Leadership Initiative on Sarcopenia (GLIS) [3].

A particularly intriguing observation in Chien et al. [1] was that participants classified as having low MSS paradoxically exhibited a greater skeletal muscle index (7.7 vs. 7.1 kg/m^2^). This finding appears counterintuitive when considered against the traditional sarcopenia concept, which links lower muscle mass with poorer outcomes. Despite this greater muscularity, these individuals demonstrated significantly lower HGS (26.1 vs. 32.0 kg). This unexpected pattern prompted us to explore potential factors underlying these discrepancies, leading to a more detailed examination of the data. Upon closer examination, we noted that, although no age differences were observed between the low‐ and high‐MSS groups, individuals with low MSS had a substantially higher BMI (26.4 vs. 23.9 kg/m^2^). This suggests that, beyond greater muscle mass, these individuals likely carried more adiposity. Consequently, their apparently higher muscle reserves were likely insufficient relative to body size and functional demands. Moreover, increased body mass—particularly excess fat—is well established as being linked to adverse metabolic and inflammatory profiles, which could partly explain the article's finding that low MSS was associated with unfavourable outcomes. Analogous to MSS calculation, HGS normalized to body mass (rHGS) also shows a negative association with body mass, BMI and waist circumference. In fact, prior studies indicate that rHGS correlates more strongly with cardiometabolic risk than absolute HGS [4], and subsequent investigations have reported that lower rHGS—but not absolute HGS—is associated with a higher prevalence of metabolic syndrome [5]. Together, these patterns suggest that the low‐MSS group may reflect a phenotype of sarcopenic obesity rather than sarcopenia per se. Clarifying these relationships—especially given that BIA‐derived body composition data appear available—could yield valuable insights into the interplay between muscle strength, adiposity and health.

HGS assessment is a simple, portable and inexpensive procedure that makes its application widely accessible, even in small health centres. Evaluating muscle mass usually requires expensive equipment (DXA, ultrasound, computed tomography and MRI), trained staff and is technically challenging to measure accurately. Moreover, variability in assessment techniques and in the specific body segments used for strength normalization (e.g., hand, forearm, arm or appendicular mass) further complicates the development of standardized cutoff values and their implementation in clinical settings. Alternatively, normalizing HGS to body mass (i.e., rHGS) may provide strong predictive value, representing a simple and practical metric to identify individuals at risk for functional decline and poor cardiometabolic health. When an older adult rises from a chair, climbs a flight of stairs or regains balance after a perturbation, their neuromuscular system must generate sufficient force to overcome gravitational demands relative to body mass. Accordingly, assessing strength solely in absolute terms may provide an incomplete picture, whereas normalizing for body mass offers a more meaningful reflection of functional demands, integrating both muscle capacity and individual requirements into a single composite metric. There is evidence that interpreting HGS relative to body mass provides a better assessment of health status than using absolute values alone [5, 6, 7, 8].

In this context, future analyses directly comparing MSS with rHGS would be of great value, and the authors appear to have already collected the data necessary to address this question. A direct comparison with MSS in this well‐characterized cohort could offer a unique opportunity to disentangle the relative contributions of lean mass–adjusted and body mass–adjusted strength metrics to functional and metabolic health in older adults. In this direction, it would be of clinical relevance to explore the use of evidence‐based cutoff values. The Sarcopenia Definition and Outcomes Consortium (SDOC) identified body mass normalized grip strength cutoffs of 0.45 for men and 0.34 for women [9], which could serve as a meaningful starting point for consolidating strength‐based thresholds in future studies. Exploring these comparisons could mark a critical step toward an ideal interpretation of HGS as a practical marker of muscle health and aging, enabling early intervention strategies aimed at disease prevention and health promotion.
